# Psychosocial distress and associated factors among pneumoconiosis patients: a cross-sectional study

**DOI:** 10.3389/fpubh.2026.1853040

**Published:** 2026-07-22

**Authors:** Jinglin Wang, Yuehui Fang, Meian Tang, Zuying Li, Ying Deng, Shengxi Li, Ya Tang, Yufei Luo, Rou Chen

**Affiliations:** 1 School of Nursing, Hunan University of Chinese Medicine, Changsha, China; 2 The First Affiliated Hospital of Changsha Medical University, Changsha, China; 3 Department of Occupational Medicine, Hunan Prevention and Treatment Institute for Occupational Diseases, Changsha, China; 4 School of Pharmacy, Hunan Vocational College of Science and Technology, Changsha, China

**Keywords:** distress thermometer, occupational health, pneumoconiosis, problem list, psychosocial distress

## Abstract

**Background and aims:**

Pneumoconiosis is a chronic pulmonary disease characterized by diminished physical function, impaired mental health, and deteriorated socioeconomic status. The study aims to evaluate the status of psychosocial distress and identify its associated factors in patients with pneumoconiosis.

**Methods:**

This cross-sectional study enrolled 369 patients with pneumoconiosis admitted to the Hunan Prevention and Treatment Institute for Occupational Diseases between July 2022 and March 2023. Psychosocial distress was assessed using the Distress Thermometer (DT) scale and the accompanying Problem List. An exploratory multivariable linear regression analysis was performed to identify factors associated with psychosocial distress, and sensitivity analyses were conducted to examine the robustness of the results.

**Results:**

All participants were men, with a mean age of 58.60 ± 7.43 years. Most of patients had low education levels, long exposure duration and one or more complications or comorbidities. The mean DT score was 5.42 ± 2.45, and 75.88% of patients reported clinically relevant psychosocial distress (DT score≥4). Breathing, insurance/financial, loss of memory/concentration were the main problems for psychosocial distress. Factors associated with psychosocial distress included advanced disease stage, comorbid diabetes, and medium education level.

**Conclusion:**

Psychosocial distress was common among the patients with pneumoconiosis included in our study. Breathing problems, financial concerns, and advanced disease progression were associated with higher levels of psychosocial distress. Future multi-center studies with probability sampling and inclusion of female patients are needed to confirm and extend these results.

## Introduction

Pneumoconiosis is the most severe occupational pulmonary disease worldwide, caused by inhalation and retention of dust particles in the lungs (1, 2). Annual deaths from the disease have exceeded 21,000 each year since 2015 (1). China bears the highest global burden of pneumoconiosis, experiencing both the greatest health loss (3) and the highest incidence rate among all countries (4) The progression of pneumoconiosis is rather slow, typically spanning a decade or longer (3, 5). Importantly, the disease continues to advance even after exposure to dust particles has ceased (6). To date, no curative is available for pneumoconiosis (3). Consequently, as the disease progresses, patients experienced reduced lung function, severe breathing problems, and decreased physical exercise tolerance (7, 8). Such physical and functional impairments can give rise to negative emotions (e.g., anxiety, fear, and depression) (9, 10) and socioeconomic challenges (e.g., financial strain, housing instability, and intimate relationship difficulties) (11). Many studied have pointed out that pneumoconiosis patients require equal attention to both their socioeconomic status and psychological well-being (11, 12). Accordingly, the psychosocial problems of pneumoconiosis patients have become a major focus of current research.

Psychosocial distress is a multifactorial unpleasant experience of psychological, social, spiritual and/or physical nature that may interfere with the ability to cope effectively with diseases, its physical symptoms, and its treatment (13). The Distress Thermometer (DT) is a brief, widely-used screening tool designed to quickly assess psychosocial distress, including an 11-point visual analog scale. If the patient’s distress level is >4, the medical team looks at the Problem List questionnaire to identify key issues of concern and asks further questions to determine the best resources to address the patient’s concerns. Originally, DT is commonly used for quickly identifying the psychosocial problems of cancer patients and giving corresponding intervention measures to improve the life quality (14–16). Due to its brief and easy to complete, the DT has been increasingly applied outside the field of cancer care (17). However, to our knowledge, no study has assessed the psychosocial distress in pneumoconiosis patients. Thus, in this study, we employed the DT Scale and its Problem List to assess the level of psychosocial distress among patients with pneumoconiosis and analyze its associated factors for intervention and rehabilitation treatment.

## Methods

### Study sample

The cross-sectional study was conducted between July 2022 and March 2023 in Hunan province, China. Patients with pneumoconiosis were recruited by convenience sampling in Hunan Prevention and Treatment Institute for Occupational Diseases. The inclusion criteria for participants were: (a) a clinical diagnosis certificate of pneumoconiosis (GBZ 70–2015) (18) (see Supplementary materials for detailed criteria), (b) can communicate normally; (c) without receiving psychological treatment; (d) no illness of vital organs such as tumor. Written informed consent was obtained from each participant before enrollment. The study was approved by the Hunan Prevention and Treatment Institute for Occupational Diseases Ethics Committee (Approval number: LS2021122401).

**Figure 1:**
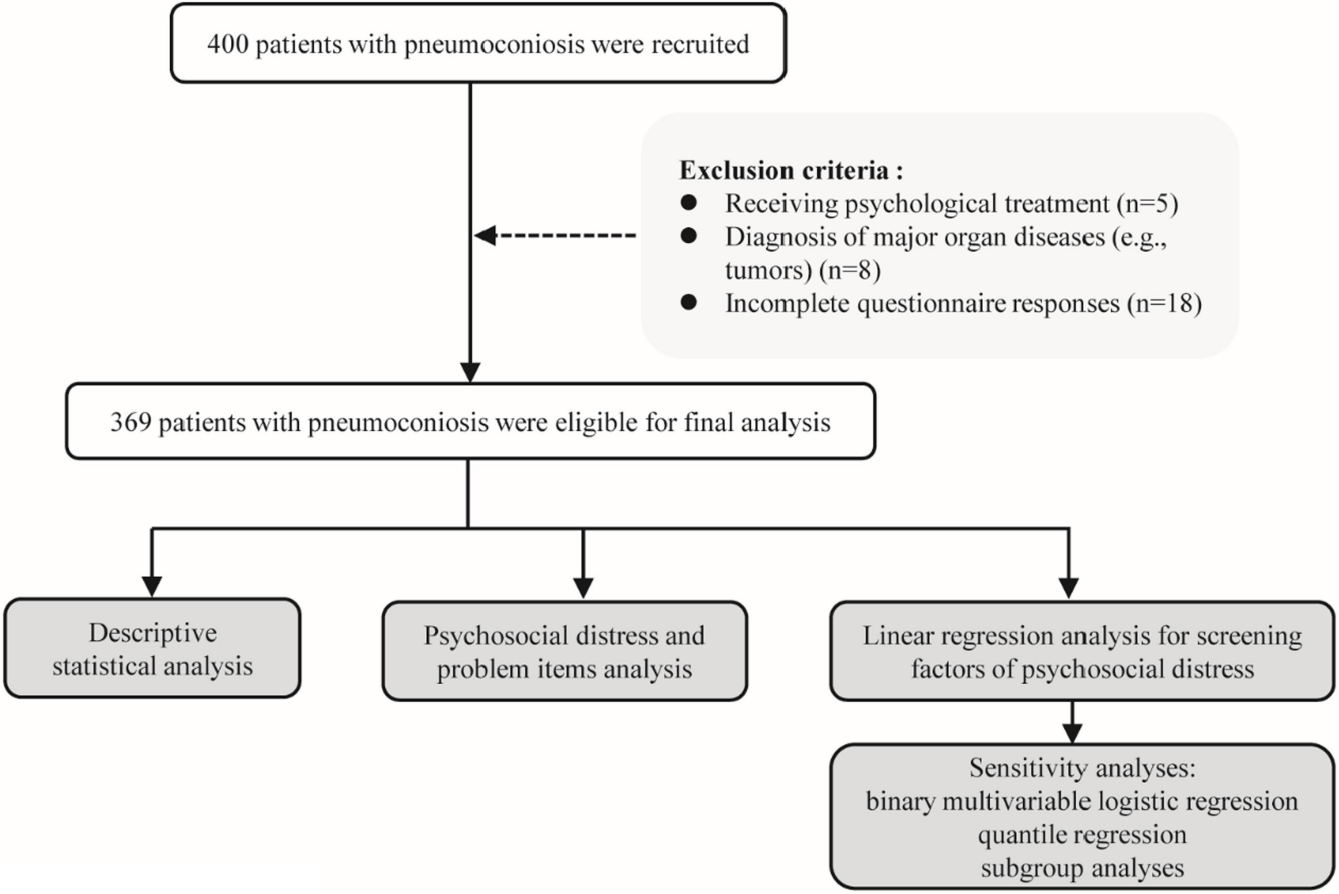
Flowchart of data collection and analyses. All enrolled patients met the criterion of normal communication ability to ensure understanding of the study purpose and procedures; those who did not were excluded before formal enrollment.

The study sample size was calculated according to Kendall’s sample estimation principle: N = independent variables× (5–10) (19, 20). There are 16 independent variables included in this study. The sample size was determined to be 90–160 cases. Besides, we also used the G-power 3.1 software to estimate sample size (21). The sample size of 295 patients was determined with a statistical power of 0.80, a significance level of 0.05, an effect size of 0.05, and 7 arbitrary factors for multivariable regression analysis (22, 23). At last, we chose the large simple size. Considering a 10% invalid questionnaire rate (295/0.9 = 328), we gave out 400 questionnaires. Three hundred sixty-nine in-hospital patients met the requirements and completed all of the questions. Thus, we use the 369 completed data for analysis. The flow chart for enrolled patients was presented in Figure 1.

### Questionnaire content

#### Sociodemographic information

The sociodemographic information was collected including two components: (a) demographic information, including age, marital status, education level, smoking status, monthly income, and annual cost of treatment; (b) disease information, including pneumoconiosis type, pneumoconiosis stage, exposure duration, exposure age and complication/comorbidity. Pneumoconiosis stage (I, II, and III) was assigned based on radiological indicators obtained from chest radiographs (see Supplementary materials for detailed criteria). The complication/comorbidity variable included both pneumoconiosis-related complications (tuberculosis, COPD, pneumothorax, respiratory failure, pulmonary atelectasis, and pulmonary heart disease) and non-respiratory comorbidities (hypertension, diabetes, and renal failure) (24). Conditions with a frequency of fewer than 10 cases were aggregated into an “other” category.

#### Distress thermometer (DT) scale and problem list (PL)

The DT consists a single-item self-report measure of psychosocial distress in the past week, which is rated on a 11-point scale that ranged from 0 to 10 (14, 25). The DT can be used for identifying distress coming from any source, even if unrelated to cancer (13, 17). More specifically, its validity in assessing psychosocial distress among patients with chronic respiratory conditions (22, 26, 27). A cutoff score of 4 was recommended by NCCN and also observed in Chinese population (28). Moreover, NCCN adds a PL to identify the sources of distress, which contains 39 items divided into 5 groups (practical problems, family/social problems, emotional problems, physical problems, spiritual/religious concerns). For each item from PL, patients only need to check YES or NO in the past week including today. The DT scale and PL are a free resource to all researchers in NCCN website^1^. PL in the Chinese version is adjusted to 40 items (28), and is presented in the Supplementary Table S1.

### Statistical analysis

Descriptive statistics were used to summarize the sociodemographic characteristics of patients. Continuous variables were represented by mean ± standard deviation, and categorical variables as frequency and percentage. Between-group comparisons of the DT score according to sociodemographic variables were performed using Student’s *t*-test (two groups) or one-way analysis of variance (three or more groups). For exploratory multivariable linear regression, we adopted a combined variable selection strategy based on previous studies (29, 30). First, a core set of variables was pre-specified based on clinical significance and published studies related to psychosocial problems among pneumoconiosis patients (age, education level, monthly income, complication/comorbidity count, COPD, and diabetes) (8); these variables were forced into the model. Second, additional candidate variables were identified through univariable analysis (*p* < 0.05). Given the DT score is a bounded 0–10 scale, ordinary linear regression does not strictly satisfy all theoretical assumptions. However, for the purpose of estimating clinically interpretable mean differences, we employed linear regression as the primary model. To verify its validity in our data, we conducted comprehensive residual diagnostics, including tests for normality and homoscedasticity (Supplementary Figure S1).

To assess the robustness of the findings, we used exploratory binary multivariable logistic regression (DT ≥ 4 vs. < 4; Supplementary Table S2) and quantile regression (at the 25th, 50th, and 75th percentiles; Supplementary Table S3) as sensitivity analyses to identify the factors associated with psychosocial distress. Additionally, we conducted exploratory subgroup analyses by age, pneumoconiosis type, and exposure duration groups and used the generalized linear model to re-examine the association between each variable and the DT score (Supplementary Tables S4–S6), with the goal of hypothesis generation rather than confirmatory testing. A two-sided *p* < 0.05 was considered statistically significant. All analyses were performed using SPSS version 27.0.

## Results

### Study population characteristics

Of 400 patients with pneumoconiosis who were recruited, 369 with complete data were included in the final analysis. The characteristics of these patients were presented in Table 1. All participants were men, with a mean age of 58.60 ± 7.43 years. The majority (94.04%) had no more than a junior high school education. Silicosis and coal workers’ pneumoconiosis accounted for 98.10% of all pneumoconiosis types. Regarding disease stage, 30.08, 26.56 and 43.56% of patients were classified as stage I, II, and III, respectively. More than half of the patients (53.93%) had one or more complications/comorbidities. Over half (53.12%) had no personal income, and 30.89% earned less than 3,000 RMB. Nevertheless, 69.65% of patients still paid for their medical treatment.

**Table 1:** Characteristics and exploratory univariable analysis of DT score in 369 patients with pneumoconiosis.

Variables	*N* (%)	DT score	*t*/*F* value	*p-*value
Age (years)				
≤50	38 (10.30)	4.95 ± 2.28	1.089	0.338
51–60	216 (58.54)	5.40 ± 2.48		
>60	115 (31.17)	5.62 ± 2.43		
Marital status				
Married	345 (93.50)	5.39 ± 2.47	0.850	0.396
Single (Unmarried, Divorced, Widowed)	24 (6.50)	5.83 ± 2.06		
Education level*				
Low	146 (39.57)	5.76 ± 2.40	4.945	0.008
Medium	201 (54.47)	5.08 ± 2.43		
High	22 (5.96)	6.32 ± 2.42		
Pneumoconiosis type				
Silicosis	156 (42.28)	5.53 ± 2.32	0.428	0.652
Coal Workers’ pneumoconiosis	206 (55.83)	5.33 ± 2.55		
Others	7 (1.90)	5.86 ± 2.41		
Pneumoconiosis stage				
I	111 (30.08)	5.06 ± 2.44	6.133	0.002
II	98 (26.56)	5.01 ± 2.53		
III	160 (43.36)	5.93 ± 2.32		
Age at onset of dust exposure (years)				
≤20	134 (36.31)	5.36 ± 2.41	0.431	0.731
21–30	141 (38.21)	5.51 ± 2.57		
31–40	71 (19.24)	5.24 ± 2.39		
>40	23 (6.23)	5.83 ± 2.08		
Exposure duration (years)				
1–10	69 (18.70)	5.52 ± 2.17	1.390	0.246
11–20	120 (32.52)	5.06 ± 2.46		
21–30	119 (32.25)	5.67 ± 2.58		
>30	61 (16.53)	5.54 ± 2.43		
Smoking status (pack-years)				
0	113 (30.62)	5.41 ± 2.36	0.338	0.853
1–10	79 (21.41)	5.22 ± 2.65		
11–20	72 (19.51)	5.50 ± 2.40		
21–30	45 (12.20)	5.36 ± 2.50		
>30	60 (16.26)	5.68 ± 2.40		
Monthly personal income (RMB)				
0	196 (53.12)	5.74 ± 2.40	3.679	0.026
1–3,000	147 (39.84)	5.06 ± 2.39		
>3,000	26 (7.05)	5.04 ± 2.85		
Annual cost of treatment (RMB)				
0	112 (30.35)	5.38 ± 2.31	1.447	0.218
1–3,000	114 (30.89)	5.06 ± 2.57		
3,001–5,000	50 (13.55)	5.54 ± 2.38		
5,001–8,000	34 (9.21)	5.74 ± 1.88		
>8,000	59 (15.99)	5.93 ± 2.74		
Complication/Comorbidity count				
0	170 (46.07)	5.38 ± 2.48	0.153	0.928
1	151 (40.92)	5.40 ± 2.41		
2	39 (10.57)	5.64 ± 2.56		
3	9 (2.44)	5.67 ± 2.29		
Complication/Comorbidity type				
Tuberculosis	47 (12.74)	5.06 ± 2.35	−1.077	0.282
COPD	84 (22.76)	5.58 ± 2.55	0.684	0.494
Diabetes	16 (4.34)	6.44 ± 2.58	1.612	0.126
Hypertension	62 (16.80)	5.26 ± 2.35	−0.581	0.562
Others	47 (12.74)	5.79 ± 2.27	1.094	0.275

*Education level was categorized as low (primary school or below), medium (junior high school or technical secondary school), and high (senior high school or above).

### The psychosocial distress in pneumoconiosis patients

**Figure 2:**
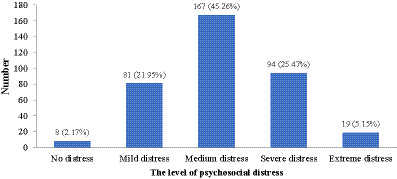
The distribution of psychosocial distress from 369 patients with pneumoconiosis. DT score is classified as five categories: no distress (score 0), mild distress (score 1–3), medium distress (score 4–6), severe distress (score 7–9), and extreme distress (score 10).

Among the 369 participants from this single institute, the mean DT score was 5.42 ± 2.45 and 75.88% experienced clinically significant psychosocial distress (DT ≥ 4, Figure 2).

Then, we counted the sum of PL to identify the sources of distress. As shown in Table 2, the top 10 problems for psychosocial distress were breathing, insurance/financial, loss of memory/concentration, chest pain, sleep, physical activity restricted, getting around, tingling in hands/feet, worry, and fatigue.

**Table 2:** The problem items of psychosocial distress for 369 patients with pneumoconiosis.

Problem list	Number	Frequency (%)
Practical problems		
No time or energy to take care of children/older adult(s)	143	38.8
No time or energy to do housework	143	38.8
Insurance/financial	250	67.8
Transportation	95	25.7
Work/school	81	22
Surrounding	30	8.1
Family/Social problems		
Dealing with children/older adult(s)	80	21.7
Dealing with partner	85	23
Dealing with friends and family	36	9.8
Dealing with medical staff	29	7.9
Emotional problems		
Depression	67	18.2
Fears	68	18.4
Loneliness	75	20.3
Nervousness	95	25.7
Loss of interest in usual activities	100	27.1
Sadness	79	21.4
Worry	160	43.4
Sleep	194	52.6
Loss of memory/ concentration	242	65.6
Physical problems		
Appearance	44	11.9
Bathing/dressing	52	14.1
Breathing	309	83.7
Changes in urination	90	24.4
Constipation	82	22.2
Diarrhea	59	16
Eating	85	23
Fatigue	148	40.1
Feeling swollen	28	7.6
Fevers	43	11.7
Getting around	171	46.3
Indigestion	91	24.7
Chest pain	205	55.6
Nausea	106	28.7
Nose dry/congested	47	12.7
Pain	147	39.8
Sexual	36	9.8
Skin dry	76	20.6
Tingling in hands/feet	170	46.1
Physical activity restricted	174	47.2
Spiritual/religious concerns	10	2.7

### The associated factors of psychosocial distress in pneumoconiosis patients

In univariate analysis, education level, pneumoconiosis stage, and monthly personal income were significantly associated with psychosocial distress (Table 1, *p* < 0.05). These variables, together with pre-specified variables based on clinical significance and published studies, were then entered simultaneously into multivariable linear regression model to examine their independent associations with psychosocial distress. Residual analysis of the linear regression model revealed no obvious deviation from normality (Supplementary Figure S1A,B) or homoscedasticity (Supplementary Figure S1C), and no systematic patterns were observed in residual plots. As shown in Table 3, patients with stage III pneumoconiosis (*B* = 0.641, *p* = 0.038) and those with diabetes (*B* = 1.319, *p* = 0.048) have higher DT scores. In contrast, patients with medium education level had significantly lower DT scores (*B* = −0.549, *p* = 0.048).

**Table 3:** Exploratory multivariable linear regression analysis of factors associated with psychosocial distress (*n* = 369).

Variables	*B*	S. E.	*β*	*t* value	95%CI	*p*-value	VIF
Constant	5.289	0.499	–	10.591	(4.307,6.271)	<0.001	
Age (years)							
≤50 (ref)							
51–60	0.310	0.427	0.063	0.726	(−0.530, 1.150)	0.468	2.859
>60	0.394	0.464	0.075	0.849	(−0.519, 1.307)	0.397	2.986
Education level							
Low (ref)							
Medium	−0.549	0.276	−0.112	−1.986	(−1.093, −0.005)	0.048	1.224
High	0.663	0.554	0.064	1.195	(−0.428,1.753)	0.233	1.113
Pneumoconiosis stage							
I (ref)							
II	−0.007	0.334	−0.001	−0.020	(−0.664,0.651)	0.984	1.408
III	0.641	0.307	0.130	2.088	(0.037,1.246)	0.038	1.496
Monthly personal income (RMB)							
0 (ref)							
≤3,000	−0.435	0.276	−0.087	−1.576	(−0.977,0.108)	0.116	1.177
>3,000	−0.461	0.519	−0.048	−0.888	(−1.483,0.560)	0.375	1.141
Complication/Comorbidity count	−0.182	0.222	−0.056	−0.818	(−0.618,0.255)	0.414	1.811
COPD	0.383	0.384	0.066	0.999	(−0.371,1.138)	0.318	1.671
Diabetes	1.319	0.666	0.110	1.981	(0.009,2.629)	0.048	1.188

*R*^2^ = 0.073, adjust *R*^2^ = 0.045, *F* = 2.559, *p* = 0.004.

CI, confidence intervals; S. E., standard error.

Sensitivity analyses partially supported the primary findings. Medium education level was significantly associated with lower odds of high distress in logistic regression (OR 0.56, 95% CI 0.32–0.99, *p* = 0.045; Supplementary Table S2) and low level of distress in quantile regression (25th and 75th percentile; Supplementary Table S3), although education-level subgroup analyses were non-significant overall. Of note, the high-education subgroup comprised only 22 participants; estimates for this subgroup are statistically unstable, with wide confidence intervals, and warrant considerable caution in interpretation. Regarding disease stage, subgroup analyses suggested that the association between stage III pneumoconiosis and DT scores tended to be stronger among coal workers’ pneumoconiosis and in those with 21–30 years of exposure (Supplementary Tables S4–S6). However, stage III pneumoconiosis did not reach statistical significance in either logistic regression or quantile regression. Nevertheless, the direction and trend of this association were consistent across both models. Similarly, subgroup analyses suggested a stronger association between diabetes and DT scores among coal workers’ pneumoconiosis, as well as patients aged ≤50 or with 1–10 years of exposure. Other analyses showed no statistically significant associated with DT.

## Discussion

The present study found a high prevalence of psychosocial distress in our sample of the hospitalized male patients with pneumoconiosis. The proportion of clinically relevant psychological distress (DT ≥ 4) was 75.88%, which is higher than that reported for other diseases, including COPD (32.1%) (22), schizophrenia (46.9%) (31), kidney disease (59.7%) (32), and HIV (73.3%) (33). Such a strikingly high burden of psychosocial distress underscores a fundamental public health imperative, and primary prevention of pneumoconiosis must remain the absolute priority. Given the irreversible fibrotic nature of the disease and the lack of curative treatments, once pneumoconiosis develops, patients are subjected to a lifetime trajectory of not only progressive physical impairment but also enduring mental health challenges (8, 11). Therefore, the most effective psychosocial intervention is, without doubt, primary prevention. This entails the rigorous enforcement of occupational dust control, regular workplace surveillance, and health education—all aimed at preventing disease onset among currently exposed workers. However, given that a large population of pneumoconiosis patients already exists and cannot benefit from primary prevention, it is equally critical to shift our focus toward tertiary prevention: specifically, the psychosocial rehabilitation and holistic care for those already diagnosed. For these individuals, the physical impairment is permanent, yet the associated psychosocial distress may be modifiable through targeted interventions. Therefore, while primary prevention is the ultimate goal for high-risk populations, for the existing hospitalized patients in our cohort, exploring the common problems and associated factors of psychosocial distress is of immediate clinical value.

According the results from PL analysis, breathing (83.7%), insurance/financial (67.8%), and memory/concentration loss (65.6%) emerged as the predominant contributors to distress. Notably, these problems are not merely physical or economic; they profoundly affect patients’ perceived self-efficacy and social integration. Pneumoconiosis is an irreversible lung disease caused by occupational exposure to dust. Fine dust particles inhaled into the alveoli can cause lung damage (10), leading to decreased lung function and other dust-related lung diseases, including COPD (34) and tuberculosis (35). Research had shown that comorbid conditions such as tuberculosis and COPD significantly worsened co-occurring depression and anxiety symptoms among pneumoconiosis patients (36–38). As lung damage progresses, respiratory symptoms, such as cough, sputum production, wheezing, chest pain, and dyspnea, typically become more severe (39). Research had shown that more severe dyspnea is correlated with worse mental health (36–38). In our study, most participants were in the advanced stage of pneumoconiosis. Among them, 23% were found to have COPD, and 13% had tuberculosis. These respiratory problems may negatively affect patients’ quality of life (40), which could in turn contribute to the development of psychosocial distress. This may explain why the breathing was the most frequently reported problem associated with psychosocial distress. Thus, breathing problem was associated with psychological distress in this study, and future research is needed to test whether strengthening respiratory rehabilitation can effectively alleviate such distress. Meantime, a decline in work capacity is common among pneumoconiosis patients due to illness. Hu W et al. reported that more than 40% of patients lost their ability to work and had difficulty engaging in productive activities (12). Most patients lack compensation coverage and still bear a heavy medical burden. As the primary breadwinners, many patients forgo treatment to avoid imposing further economic hardship on their households (12), while concurrently experiencing guilt over their inability to contribute financially. This economic strain thus emerges as a major driver of psychosocial distress. Accordingly, we recommended that social institutions can provide appropriate re-employment opportunities and that the government increase employment subsidies for individuals with pneumoconiosis. Regarding impaired concentration, this is more likely attributable to breathing problems that affect cerebral function, leading to deficits in comprehension and memory. This, in turn, results in a reduced quality of life and could further contribute to psychosocial distress.

Furthermore, we conducted an exploratory multivariable linear regression analysis to identify the socio-demographic and clinical determinants of this distress. The results showed that pneumoconiosis stage, education level and comorbid diabetes were associated with patients’ psychosocial distress. Patients with pneumoconiosis stage III had higher DT scores compared with other pneumoconiosis stages (5.93 ± 2.32 in stage III, 5.01 ± 2.44 in stage II and 5.06 ± 2.53 in stage I). Subgroup analyses also suggested that the association between stage III pneumoconiosis and DT scores tended to be stronger among coal workers’ pneumoconiosis and in those with 21–30 years of exposure. However, stage III pneumoconiosis did not reach statistical significance for clinically relevant distress (DT ≥ 4) in logistic regression. Notably, the direction and trend of the association between pneumoconiosis stage and DT were consistent across both models. This discrepancy is attributable to fundamental differences between the two statistical methods. Linear regression treats the outcome as a continuous variable, capturing subtle changes in DT scores across the full 0–10 range. In contrast, logistic regression dichotomizes DT into two categories, which inevitably leads to information loss and reduced statistical power. A post-hoc power analysis for the binary outcome (DT ≥ 4) revealed a statistical power of 0.352 (*α* = 0.05), with a small between-group effect size (Cohen’s *h* = 0.1935). Thus, the current sample size was inadequate to reliably detect such a small effect. Taken together, the non-significant result in the logistic regression should be interpreted with caution. A previous study has shown that patients at higher stages of pneumoconiosis tend to exhibit lower psychological well-being (11). In advanced stage, breathing difficulties worsen alongside physical problems and complications/comorbidities, leading to markedly reduced quality of life. These changes further lead to emotion problem such as anxiety, fear, depression, and low self-esteem. Consequently, a vicious cycle develops in which poor physical health and poor mental health mutually reinforce each other, with the latter adversely affecting the management of physical conditions (41). Therefore, medical staff and public health officials should pay close attention to the psychosocial problems at the time of pneumoconiosis diagnosis.

In addition, our study showed that patients with a medium education level had lower DT score (5.76 ± 2.40 for low education, 5.08 ± 2.43 for medium education, and 6.32 ± 2.42 for high education). The multivariable logistic regression also indicated medium education level was associated with lower psychosocial distress. Among low- and high-education patients, stage III pneumoconiosis was more common (48.6 and 50.0%, respectively) than medium-education patients (38.8%). Similarly, no-income status was more frequent in low- and high-education groups (65.1 and 50.0%) than in the medium group (44.8%). Studies showed that pneumoconiosis patients with lower education level are likely to have lower psychological well-being (11, 42). These patients often lack disease knowledge, have low income, and face high treatment costs. In our study, 76.7% of patients with low education had annual treatment costs exceeding 3,000 RMB, compared with 65.58% for those with medium education and 38.75% for those with high education. These barriers prevent them from seeking timely psychosocial support. Studies also link higher education to mental health distress (43–45). Our exploratory subgroup analyses suggested a similar association in participants with silicosis. However, this subgroup comprised only 22 individuals; therefore, any findings involving this subgroup are statistically unstable and should be interpreted with great caution. Thus, our primary conclusion regarding education is limited to the medium-education association observed in the main analysis. In addition, pneumoconiosis patients with comorbid diabetes had significantly higher DT scores in our analysis. Subgroup analyses partially aligned with this observation, with consistent directional trends across both logistic and quantile regression models. However, neither regression model yielded statistically significant results for this association. A plausible explanation for this discrepancy is the extremely limited sample size of the diabetic subgroup, which comprised only 16 patients, yielding unstable estimates with wide confidence intervals that preclude robust inferential conclusions. Previous research has demonstrated that individuals with diabetes commonly experience diabetes-related distress (46, 47), and that diabetes induces structural changes in the lungs and exacerbates the impairment of lung function (48). Thus, the additional physical burden of diabetes may increase these patients’ susceptibility to psychosocial distress, suggesting the clinical value of integrating routine blood glucose monitoring into pneumoconiosis management.

The study has several limitations. First, the generalizability of our findings is limited by the sampling strategy. Participants were recruited using convenience sampling from a single occupational disease prevention institute in Hunan Province, and all were male. This all-male sample reflects the current epidemiology of pneumoconiosis in China (where most affected individuals are men) (49), but our findings cannot be directly extrapolated to female population; if women experience or report distress differently, the direction of bias is uncertain. In addition, recruitment from a single institution in Hunan may not represent patients from other provinces with different industrial exposures, healthcare systems, or socioeconomic conditions. Moreover, convenience sampling may have introduced volunteer bias: distressed individuals might be more willing to participate (potentially overestimating distress levels), while those with severe illness or lower health literacy might be underrepresented (potentially underestimating distress). Thus, future multi-center studies with probability sampling and inclusion of female patients are needed to confirm and extend our results. Second, this study explained only 4.5% of the variance in psychosocial distress, suggesting that most contributors remain unmeasured. Potential unassessed confounders included dyspnea severity, lung function, time since diagnosis, disability grade, compensation status, pain severity, sleep quality, oxygen therapy, time since cessation of dust exposure, previous psychiatric history, social support, personality traits, and healthcare access. Notably, we lacked objective measures of respiratory severity (e.g., pulmonary function tests, arterial blood gas analysis), which is especially concerning because breathing difficulty was the most frequently reported problem among our participants. This could prevent us from distinguishing between perceived symptom burden and actual physiological impairment. Thus, our respiratory-related findings are tentative, and future studies should incorporate both objective pulmonary assessments and broader biopsychosocial measures to confirm these associations. Our results should be seen as identifying potential correlates, not as a full model of distress factors. Third, several limitations regarding the measurement of socioeconomic status using monthly personal income should be acknowledged. More than half (53.1%) of our patients reported zero monthly personal income. This high proportion reflects a study population consisting largely of retired, unemployed, or informally employed individuals, many of whom rely on family support, pensions, or non-cash resources. Consequently, zero personal income is a heterogeneous category rather than an indicator of no economic resources. Future research should employ more comprehensive measures of financial status, such as household per capita income, subjective financial strain, or material assets, to better capture the economic circumstances of patients with pneumoconiosis. Additionally, using absolute income in the local currency (RMB) limits cross-regional comparability and fails to adequately reflect participants’ relative socioeconomic status. To enhance scientific rigor and generalizability, future research should classify income levels relative to local medians or population-based percentiles (e.g., low/middle/high). Fourth, we excluded patients who were already receiving psychological treatment at the time of enrollment. These patients may have had more severe psychological distress or, conversely, better-managed distress. Because we cannot determine which effect dominates, our findings should be interpreted as applying only to pneumoconiosis inpatients not currently engaged in psychological therapy. Future studies that include patients receiving psychological care are needed to assess the generalizability of our results to the full patient population. Finally, the cross-sectional nature of our data does not allow for causal interpretation. Future longitudinal cohort studies are warranted to examine the temporal direction of these relationships, and interventional trials would be required to test whether improving respiratory rehabilitation or financial support reduces distress.

## Conclusion

Our findings from this hospitalized male cohort indicate that elevated psychosocial distress was common among patients with pneumoconiosis, particularly in those with advanced disease stages, breathing difficulty, and financial insecurity. A tentative association between medium-level education and lower distress was also noted, although this finding should be interpreted with caution given the small sample size in the higher-education subgroup. Collectively, these results carry dual implications. For workers currently exposed to dust, these findings reinforce the urgent need for stringent primary prevention to avert the disease entirely. For the substantial population already living with pneumoconiosis, our results underscore the importance of integrating psychosocial screening and targeted psychosocial support into routine clinical care, with particular attention to disease severity, symptom burden, and socioeconomic vulnerability.

## Data Availability

The datasets presented in this study can be found in online repositories. The names of the repository/repositories and accession number(s) can be found in the article/Supplementary material.

## Footnotes

1
https://www.nccn.org
