# Supplementary material for: Psychosocial distress and associated factors among pneumoconiosis patients: a cross-sectional study
Source: Front Public Health. 2026 Jul 22;14:1853040. doi: 10.3389/fpubh.2026.1853040 (PMC13438197; doi:10.3389/fpubh.2026.1853040)
Supplement: Supplementary file 1 [file Data_Sheet_1.pdf]

# Supplementary materials

## Diagnostic criteria for pneumoconiosis

All pneumoconiosis patients enrolled in this study were diagnosed strictly in accordance with the national occupational health standard Diagnosis of Occupational Pneumoconiosis (GBZ 70-2015), which is consistent with the ILO International Classification of Radiographs of pneumoconiosis. The diagnosis workflow was standardized as follows:

(a) The subjects had a reliable history of long-term occupational exposure to mineral dusts;

(b) Standard high-kilovolt chest radiographs were obtained for each participant; two radiologists specialized in pneumoconiosis diagnosis independently evaluated all imaging images;

(c) We collected field occupational hygiene survey data and longitudinal imaging follow-up records;

(d) Confounding diseases mimicking pneumoconiosis (e.g., pulmonary tuberculosis, primary lung cancer, idiopathic pulmonary fibrosis, sarcoidosis, bronchiectasis) were fully excluded;

(e) Formal clinical pneumoconiosis diagnostic certificates issued by qualified occupational disease diagnostic institutions (Hunan Prevention and Treatment Institute for Occupational Diseases) were available for all participants.

## Staging classification (Stage I, II, III) and adopted parameters

Consistent with Chapter 5 of GBZ 70-2015, only radiological indicators derived from high-kilovolt chest radiographs were adopted for staging, including two core parameters: the overall profusion grade of small opacities and the number of involved lung zones. Clinical manifestations and pulmonary function (physiological parameters) were not used to define disease stages; these indicators were only applied to analyze the correlation with psychosocial distress in our statistical analysis.

Detailed staging definitions:

Stage I: Small opacities of profusion grade 1 distributed over no less than 2 lung zones;

Stage II: Either (a) small opacities of profusion grade 2 covering ≥4 lung zones, or (b) small opacities of profusion grade 3 distributed across exactly 4 lung zones;

Stage III: Any one of the following conditions:

① Large opacities exist with a long diameter ≥20 mm and a short diameter >10 mm;

② Grade 3 small opacities distributed in more than 4 lung zones accompanied by aggregation of small opacities;

③ Grade 3 small opacities distributed in more than 4 lung zones together with large opacities;

④ For asbestos-exposed individuals, grade 3 small opacities combined with fused pleural plaques.

**TABLE S1 The DT and PL item list for Chinese version (40 items)**.

下面请选择最符合您**近一周**所经历的平均痛苦水平，在方格中画 “√”。

**没有痛苦** → **极度痛苦**

| 0 | 1 | 2 | 3 | 4 | 5 | 6 | 7 | 8 | 9 | 10 |
|---|---|---|---|---|---|---|---|---|---|---|
| | | | | | | | | | | |

以下哪些选项是引起您痛苦的原因，请您根据自己的实际情况在相应的选项中打“√”。

| **问题列表** | **Problem List** |
|---|---|
| **实际问题** | **Practical problems** |
| 无时间精力照顾孩子/老人 | No time or energy to take care of children/elderly |
| 无时间精力做家务 | No time or energy to do housework |
| 经济问题 | Insurance/financial |
| 交通出行 | Transportation |
| 工作/上学 | Work/school |
| 周围环境 | Surrounding |
| **交往问题** | **Family/Social problems** |
| 与孩子/老人相处 | Dealing with children/elderly |
| 与伴侣相处 | Dealing with partner |
| 与亲友相处 | Dealing with friends and family |
| 与医护人员相处 | Dealing with medical staff |
| **情绪问题** | **Emotional problems** |
| 抑郁 | Depression |
| 恐惧 | Fears |
| 孤独 | Loneliness |
| 紧张 | Nervousness |
| 对日常活动丧失兴趣 | Loss of interest in usual activities |
| 悲伤 | Sadness |
| 担忧 | Worry |
| 睡眠 | Sleep |
| 记忆力下降/注意力不集中 | Loss of memory/ concentration |
| **身体问题** | **Physical problems** |
| 外表/形体 | Appearance |
| 洗澡/穿衣 | Bathing/dressing |
| 呼吸 | Breathing |
| 排尿改变 | Changes in urination |
| 便秘 | Constipation |
| 腹泻 | Diarrhea |
| 进食 | Eating |

| | |
|---|---|
| 疲乏 | Fatigue |
| 水肿 | Feeling swollen |
| 发热 | Fevers |
| 头晕 | Getting around |
| 消化不良 | Indigestion |
| 胸痛 | Chest pain |
| 恶心 | Nausea |
| 鼻腔干燥/充血 | Nose dry/congested |
| 疼痛 | Pain |
| 性 | Sexual |
| 皮肤干燥 | Skin dry |
| 手脚麻木 | Tingling in hands/feet |
| 身体活动受限 | Physical activity restricted |
| **宗教信仰问题** | **Spiritual/religious concerns** |

Explanation: In practical problems, transportation represents whether the patient has problem with normal travel, and work/school refers to whether the patient has problems performing their occupational or educational duties due to their health condition such as reduced productivity or frequent absences. In physical problems, appearance refers to whether the patient's appearance changes due to the influence of the illness. In emotional problems, depression represents whether the patient has feelings of low mood, slow thinking or reduced activity, and nervousness refers to whether the patient experiences mental tension, unease and restlessness, accompanied by physiological reactions such as accelerated heartbeat, tremors, sweating, and restlessness.

**TABLE S2 Exploratory multivariable logistic regression analysis of factors associated with clinically significant distress (DT ≥ 4) among 369 pneumoconiosis patients.**

| Variables | Unadjusted analysis | | | Adjusted analysis | | |
|---|---|---|---|---|---|---|
| | OR | 95%CI | *p* value | OR | 95%CI | *p* value |
| **Age (years)** | | | | | | |
| ≤50 | 1.00 | (ref) | | 1.00 | (ref) | |
| 51-60 | 1.07 | 0.49-2.35 | 0.863 | 0.82 | 0.36-1.90 | 0.645 |
| >60 | 1.29 | 0.55-3.00 | 0.561 | 0.75 | 0.30-1.92 | 0.552 |
| **Marital status** | | | | | | |
| Married | 1.00 | (ref) | | | | |
| Single (Unmarried, Divorced, Widowed) | 2.32 | 0.68-7.98 | 0.180 | | | |
| **Education level** | | | | | | |
| Low | 1.00 | (ref) | | 1.00 | (ref) | |
| Medium | 0.52 | 0.31-0.88 | 0.014 | 0.56 | 0.32-0.99 | 0.045 |
| High | 0.98 | 0.31-3.12 | 0.966 | 1.11 | 0.32-3.82 | 0.871 |
| **Pneumoconiosis type** | | | | | | |
| Silicosis | 1.00 | (ref) | | | | |
| Coal Workers' pneumoconiosis | 0.78 | 0.48-1.28 | 0.332 | | | |
| Others | 1.67 | 0.20-14.37 | 0.639 | | | |
| **Pneumoconiosis stage** | | | | | | |
| I | 1.00 | (ref) | | 1.00 | (ref) | |
| II | 0.70 | 0.38-1.27 | 0.237 | 0.73 | 0.39-1.38 | 0.335 |
| III | 1.52 | 0.85-2.74 | 0.160 | 1.48 | 0.79-2.77 | 0.225 |
| **Age at onset of dust exposure (years)** | | | | | | |
| ≤20 | 1.00 | (ref) | | | | |
| 21-30 | 0.48 | 0.13-1.71 | 0.257 | | | |
| 31-40 | 0.39 | 0.11-1.40 | 0.148 | | | |
| > 40 | 0.56 | 0.15-2.14 | 0.397 | | | |
| **Exposure duration (years)** | | | | | | |
| 1-10 | 1.00 | (ref) | | 1.00 | (ref) | |
| 11-20 | 0.47 | 0.23-0.98 | 0.045 | 0.37 | 0.17-0.79 | 0.010 |
| 21-30 | 0.65 | 0.31-1.38 | 0.266 | 0.59 | 0.27-1.27 | 0.175 |
| > 30 | 0.96 | 0.39-2.36 | 0.924 | 0.81 | 0.32-2.07 | 0.654 |
| **Smoking status (pack-years)** | | | | | | |
| 0 | 1.00 | (ref) | | | | |
| 1-10 | 1.08 | 0.50-2.33 | 0.840 | | | |
| 11-20 | 0.56 | 0.26-1.22 | 0.146 | | | |
| 21-30 | 0.89 | 0.39-2.03 | 0.791 | | | |
| > 30 | 0.97 | 0.38-2.46 | 0.946 | | | |
| **Monthly personal income (RMB)** | | | | | | |
| 0 | 1.00 | (ref) | | 1.00 | (ref) | |
| 1-3000 | 0.60 | 0.36-1.00 | 0.048 | 0.70 | 0.41-1.22 | 0.211 |

| | | | | | | |
|---|---|---|---|---|---|---|
| > 3000 | 0.45 | 0.19-1.10 | 0.080 | 0.45 | 0.17-1.19 | 0.109 |
| **Annual cost of treatment (RMB)** | | | | | | |
| 0 | 1.00 | (ref) | | | | |
| 1-3000 | 0.89 | 0.49-1.62 | 0.708 | | | |
| 3001-5000 | 1.06 | 0.49-2.30 | 0.892 | | | |
| 5001-8000 | 1.56 | 0.58-4.15 | 0.377 | | | |
| > 8000 | 1.31 | 0.61-2.81 | 0.494 | | | |
| **Complication/ Comorbidity count** | 1.20 | 0.86-1.67 | 0.278 | 1.25 | 0.78-1.98 | 0.355 |
| **Complication/Comorbidity type** | | | | | | |
| Tuberculosis | 1.05 | 0.51-2.15 | 0.902 | | | |
| COPD | 0.94 | 0.54-1.65 | 0.830 | 0.68 | 0.32-1.48 | 0.334 |
| Diabetes | 2.29 | 0.51-10.27 | 0.280 | 2.13 | 0.42-10.86 | 0.365 |
| Hypertension | 1.24 | 0.64-2.41 | 0.525 | | | |
| Others | 1.64 | 0.74-3.65 | 0.227 | | | |

HL test: $p$ =0.496. AUC: 0.676 (95%CI: 0.613-0.738, $p < 0.001$).

**Table S3 Quantile regression analysis of factors associated with psychosocial distress.**

| Variables | QR 0.25 | | | QR 0.50 | | | QR 0.75 | | |
|---|---|---|---|---|---|---|---|---|---|
| | **β** | **S.E.** | ***p*** | **β** | **S.E.** | ***p*** | **β** | **S.E.** | ***p*** |
| **Age (years)** | | | | | | | | | |
| ≤50 (ref) | | | | | | | | | |
| 51-60 | -0.333 | 0.605 | 0.582 | 3.26E-16 | 0.562 | 1.000 | 0.800 | 0.605 | 0.187 |
| >60 | -0.333 | 0.658 | 0.613 | 0.500 | 0.611 | 0.414 | 1.200 | 0.658 | 0.069 |
| **Education level** | | | | | | | | | |
| Low (ref) | | | | | | | | | |
| Medium | -1.000 | 0.392 | 0.011 | -0.500 | 0.364 | 0.170 | -0.800 | 0.392 | 0.042 |
| High | 0.333 | 0.786 | 0.672 | 1.000 | 0.730 | 0.171 | -0.200 | 0.786 | 0.799 |
| **Pneumoconiosis stage** | | | | | | | | | |
| I (ref) | | | | | | | | | |
| II | -0.333 | 0.474 | 0.482 | -4.28E-17 | 0.440 | 1.000 | -6.94E-17 | 0.474 | 1.000 |
| III | 0.667 | 0.435 | 0.127 | 0.500 | 0.404 | 0.217 | 0.400 | 0.435 | 0.359 |
| **Monthly personal income** | | | | | | | | | |
| 0 (ref) | | | | | | | | | |
| ≤3000 | -0.667 | 0.391 | 0.089 | -0.500 | 0.363 | 0.169 | -0.600 | 0.391 | 0.126 |
| > 3000 | -1.333 | 0.736 | 0.071 | -1.000 | 0.684 | 0.144 | 0.600 | 0.736 | 0.416 |
| **Complication/ Comorbidity count** | 0.000 | 0.314 | 1.000 | -3.26E-16 | 0.292 | 1.000 | -0.600 | 0.314 | 0.057 |
| **COPD** | 0.333 | 0.544 | 0.540 | 7.59E-16 | 0.505 | 1.000 | 1.400 | 0.544 | 0.010 |
| **Diabetes** | 1.667 | 0.944 | 0.078 | 4.13E-16 | 0.877 | 1.000 | 1.800 | 0.944 | 0.057 |
| $R^2$ (adjusted $R^2$) | | 0.065 | | | 0.048 | | | 0.066 | |

**TABLE S4 Exploratory subgroup analysis by age.**

| Subgroup | Age (years) | | |
|---|---|---|---|
| | ≤50 | 51-60 | >60 |
| **Education level** | | | |
| Low (ref) | | | |
| Medium | -0.085(-1.564,1.394) | -0.415(-1.124,0.295) | -0.791(-1.678,0.097) |
| High | 1.937(-1.001,4.875) | 0.324(-1.097,1.744) | 1.401(-0.514,3.316) |
| **Pneumoconiosis stage** | | | |
| I (ref) | | | |
| II | -0.922(-2.450,0.607) | 0.141(-0.751,1.033) | 0.078(-1.055,1.211) |
| III | 0.585(-0.975,2.146) | 0.766(-0.018,1.550) | 0.504(-0.532,1.540) |
| **Monthly personal income (RMB)** | | | |
| 0 (ref) | | | |
| 1-3000 | -0.993(-2.457,0.472) | -0.167(-0.894,0.559) | -0.717(-1.619,0.186) |
| > 3000 | 0.249(-1.520,2.018) | -1.150(-2.487,0.187) | 1.765(-0.667,4.197) |
| **Diabetes** | 4.148(0.114,8.182)* | 0.643(-1.336,2.621) | 0.833(-0.814,2.480) |

*$p < 0.05$

**TABLE S5 Exploratory subgroup analysis by pneumoconiosis type.**

| Subgroup | Pneumoconiosis type | |
|---|---|---|
| | Silicosis | Coal Workers' pneumoconiosis |
| **Education level** | | |
| Low (ref) | | |
| Medium | -0.628(-1.362,0.105) | -0.363(-1.078,0.352) |
| High | 1.767(0.028,3.506)* | 0.205(-1.148,1.599) |
| **Pneumoconiosis stage** | | |
| I (ref) | | |
| II | -0.730(-1.711,0.251) | 0.479(-0.376,1.335) |
| III | -0.089(-0.913,0.735) | 1.283(0.455,2.111)* |
| **Monthly personal income (RMB)** | | |
| 0 (ref) | | |
| 1-3000 | -0.634(-1.426,0.157) | -0.170(-0.897,0.557) |
| > 3000 | -0.049(-1.665,1.567) | -0.899(-2.275,0.477) |
| **Diabetes** | 0.203(-1.584,1.990) | 1.759(0.128,3.391)* |

*$p < 0.05$

**TABLE S6 Exploratory subgroup analysis by exposure duration.**

| Subgroup | Exposure duration (years) | | | |
|---|---|---|---|---|
| | 1-10 | 11-20 | 21-30 | > 30 |
| **Education level** | | | | |
| Low (ref) | | | | |
| Medium | 0.526 (-0.626,1.678) | -1.047 (-1.925,-0.169)* | -0.107 (-1.056,0.843) | -0.924 (-2.056,0.208) |
| High | 1.676 (-0.139,3.492) | -0.093 (-2.318,2.133) | 0.883 (-0.806,2.572) | 1.973 (-2.568,6.513) |
| **Pneumoconiosis stage** | | | | |
| I (ref) | | | | |
| II | -1.022 (-2.245,0.200) | 0.199 (-1.042,1.439) | 0.803 (-0.344,1.950) | -1.172 (-2.682,0.338) |
| III | -0.688 (-1.796,0.419) | 0.812 (-0.232,1.856) | 1.417 (0.327,2.506)* | 0.560 (-0.850,1.970) |
| **Monthly personal income (RMB)** | | | | |
| 0 (ref) | | | | |
| 1-3000 | -0.963 (-1.969,0.043) | -0.069 (-1.031,0.893) | -0.540 (-1.503,0.423) | -0.886 (-2.206,0.433) |
| > 3000 | -0.986 (-2.777,0.804) | -0.900 (-2.931,1.131) | -1.271 (-3.151,0.608) | 0.137 (-1.788,2.061) |
| **Diabetes** | 4.519 (1.711,7.328)* | 1.241 (-0.890,3.372) | 1.182 (-1.038,3.401) | -0.753 (-3.029,1.523) |

Note: These subgroup analyses (by age, pneumoconiosis type, and exposure duration) are exploratory only. Multivariable subgroup comparisons were performed without adjustment for multiple testing. No formal interaction tests were conducted between subgroup factors and the exposure. Therefore, the results should be interpreted with caution and are considered hypothesis-generating rather than confirmatory. *$p < 0.05$

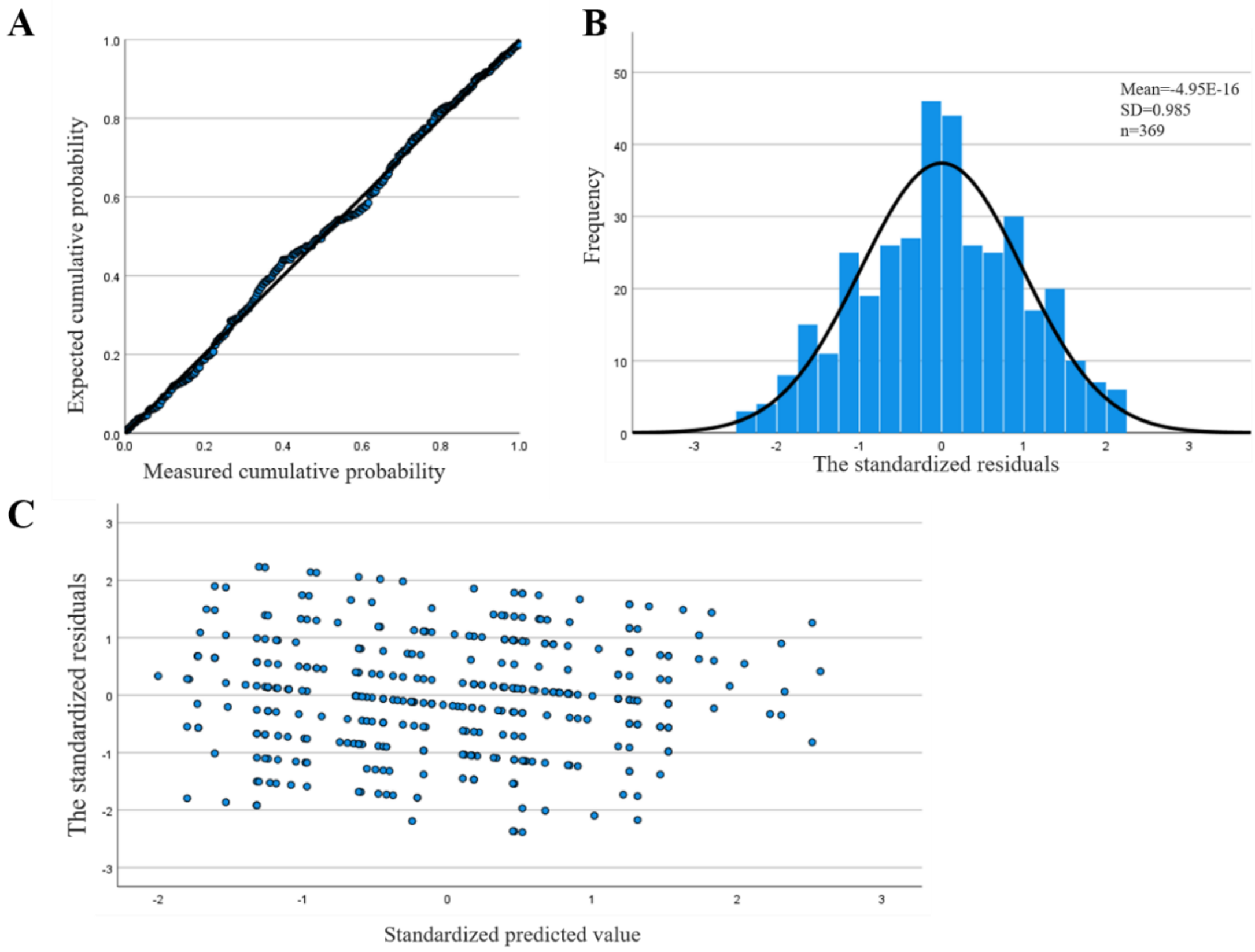


**FIGURE S1**

The residual diagnostics for exploratory multivariable linear regression. A, Normal P-P plot of standardized residuals. B, The Histogram of standardized residuals. C, The Scatter plot of standardized residuals for homoscedasticity.
